# Developmental finite element analysis of cichlid pharyngeal jaws: Quantifying the generation of a key innovation

**DOI:** 10.1371/journal.pone.0189985

**Published:** 2018-01-10

**Authors:** Tim Peterson, Gerd B. Müller

**Affiliations:** 1 Department of Theoretical Biology, University of Vienna, Wien, Austria; 2 The KLI Institute, Klosterneuburg, Austria; Universiteit Gent, BELGIUM

## Abstract

Advances in imaging and modeling facilitate the calculation of biomechanical forces in biological specimens. These factors play a significant role during ontogenetic development of cichlid pharyngeal jaws, a key innovation responsible for one of the most prolific species diversifications in recent times. MicroCT imaging of radiopaque-stained vertebrate embryos were used to accurately capture the spatial relationships of the pharyngeal jaw apparatus in two cichlid species (*Haplochromis elegans* and *Amatitlania nigrofasciata*) for the purpose of creating a time series of developmental stages using finite element models, which can be used to assess the effects of biomechanical forces present in a system at multiple points of its ontogeny. Changes in muscle vector orientations, bite forces, force on the neurocranium where cartilage originates, and stress on upper pharyngeal jaws are analyzed in a comparative context. In addition, microCT scanning revealed the presence of previously unreported cement glands in *A*. *nigrofasciata*. The data obtained provide an underrepresented dimension of information on physical forces present in developmental processes and assist in interpreting the role of developmental dynamics in evolution.

## Introduction

Development utilizes both genetically controlled and context-dependent cues, such as generic cell and tissue interactions with the physiochemical environment and interactions among tissues themselves [1–4]. These non-programmed aspects of development are called upon by the developmental system based on local signals and can result in changes across the entire organism or remain confined to distinct parts. Embryogenesis therefore is modularly organized, dependent on the interactions of global patterning mechanisms and local stimuli to generate the phenotype.

An often-neglected aspect of development that is present in most multicellular organisms is mechanotransduction. Biomechanical forces are also common cues in development. The induction of bone or cartilage has been found to be caused by mechanical tension and compression, and biomechanical forces can also have a role in altering structures in the adult phenotype, such as the remodeling of bone [5–10]. The importance of biomechanical factors in development has been covered in several books [11,12], in a general overview [13], and in a multitude of papers on the biomechanics of structural elements [1,14–16]. Therefore, as the developmental system is a mediator of phenotypic evolution, and the biomechanical forces present during embryogenesis influence developmental change, an increase in knowledge about the spatial configurations and force production during development would be beneficial to understanding the evolution of phenotypic structures that, until now, have been studied from purely genetic or histological perspectives.

Modeling has become a powerful tool in understanding the physical forces in biological systems. A small sample of the organisms modeled shows a wide variety of topics, including the human knee [17], insect locomotion [18,19], unicellular mechanics [20], plant growth [21], neurulation [22], gastrulation [23], heart development [24], and many evolutionary studies focus on the skull and feeding apparatus [25–42]. A fundamental problem with creating physical models of developing elements is that the irregularly curved and complex three-dimensional structures found in living organisms do not easily translate to mathematical computation. Transfer of forces and their influence on shape changes is straightforward in objects shaped as two-dimensional plates or three-dimensional tetrahedrons, but structures in animals rarely conform to such regular shapes, and models that precisely reflect specimens’ morphology are needed.

Engineers working with mechanical forces in complex systems use a method known as finite element analysis (FEA) to overcome the problems involved with complex shapes. FEA is a technique to simulate how the physical components of a system will behave under various restraint and loading conditions. In FEA the structure of interest is divided into a large but finite number of small shapes (or “elements”) whose reactions to mechanical forces are known. These elements can have various forms, but are usually beams, plates, tetrahedrons, or hexahedrons, and are connected to each other at their nodes. Each element is assigned material properties that govern how it reacts to mechanical forces, for example by deformation as controlled by the Young’s modulus and Poisson’s ratio. These material properties are based on experimental data. Forces are then assigned to selected elements in order to simulate various loading conditions. The model is usually restrained at some of its nodes in order to simulate physical constraints and to prevent it from unrealistic behavior or “flying away.” However, it is possible to have a net force of 0 N over the entire model and to prevent unrealistic motion with careful force placement.

Though FEA was created to study the forces present in a wide range of machines, in recent times biologists have increasingly adopted this technique to study the biomechanical forces at work in living organisms. This involves a wide variety of subjects such as plant seeds [43], dinosaur tails [44,45], gastropod shells [46], and insect wings [19]. Vertebrate skulls are typical structures that are frequently studied [25–42], particularly as they relate to stresses on the bones during feeding. FEA is most often used to study a single species, though a few studies have used inter- and intraspecific specimens for a comparative analysis [26,27,31,41,47].

Most FEA studies have examined adult phenotypes, with only a small sampling using FEA during ontogeny. While the precise age of a specimen is not of particular concern when working with adult phenotypes, when comparing multiple species during development, the stage must be carefully chosen based on the structures to be examined, since in most cases the timing for the developmental onset of a structure will vary between the groups. The present paper shows how FEA during ontogeny can be aided by the use of a contrasting agent and the selection of a time series, which we refer to as developmental FEA or “devFEA”. This method is used to create a set of biomechanical models during the development of a key innovation: the derived pharyngeal jaw apparatus found in cichlids.

### The cichlid pharyngeal jaw apparatus

**Fig 1:**
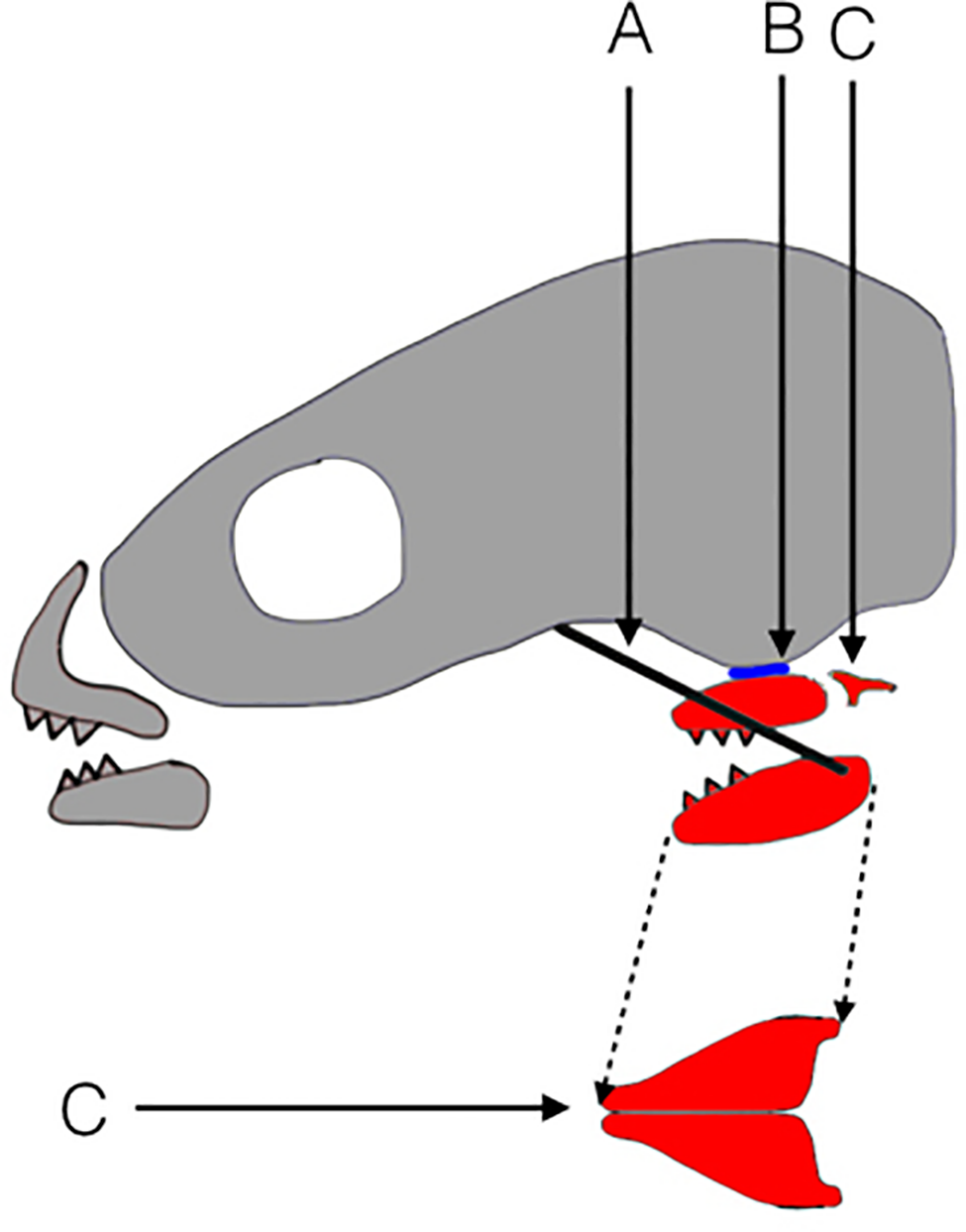
Schematic of the pharyngeal jaw apparatus of Cichlidae, adapted from Mabuchi et al 2007 [52]. Cranium and oral jaws are shaded grey. Pharyngeal jaws are shaded red. A) The black line represents the new muscle sling connecting the lower pharyngeal jaw to the neurocranium. B) The blue area marks the location of the novel basipharyngeal joint between the upper pharyngeal jaws and the neurocranium. C) The epibranchials 4 are decoupled from the upper pharyngeal jaws. Space between the two structures indicates decoupling, and does not represent their physiological distance D) Ventral view of the lower pharyngeal jaws. The two sides have fused together along the midline.

The present paper compares the biomechanics of development in two species of fish for an often-cited example of an evolutionarily important trait: The pharyngeal jaw apparatus in the Cichlidae family (Fig 1), and examines the potential and difficulties of using devFEA to expose how development and evolution are impacted by the biomechanics present during ontogeny. The pharyngeal jaw apparatus is a second set of jaws in fish derived from the pharyngeal arches. These jaws differ between species, but typically are composed of two lower, tooth-bearing ceratobranchials that bite against either a grinding plate or against two upper teeth-bearing infrapharyngobranchials. In Cichlidae the pharyngeal jaw apparatus contains a set of novelties not seen in most other fish: A) a decoupling of the epibranchials 4 from the upper pharyngeal jaws (UPJ); B) a fusion of the lower pharyngeal jaws (LPJ) creating a single functional unit C) a diarthrotic joint between the ventral side of the neurocranium and the UPJ; D) a muscle sling running from the neurocranium to the ceratobranchials 5, comprised of the *levator externus 4* and in some species the *levator posterior*; [48–50]. It has been claimed that these traits increase bite force due to the new muscle sling [48], reinforce the structural integrity of the LPJ [51], give increased range of control of the LPJ [48], and use the skull base to support the bite force [50].

The combined effect of these unique morphological features makes the pharyngeal jaw apparatus an effective food-processing unit. This has created two independently evolving modules: the oral jaws specializing in prey acquisition, and the pharyngeal jaw apparatus specializing in prey processing [48,50,53], leading the novelty to be described as a “key innovation" [54] responsible for the diversification of the cichlids [48,50,53,55]. The pharyngeal jaw apparatus is a highly plastic system which can be remodeled as a response to local stimuli [56–59], and it has been suggested that aspects of it could have formed due to mechanical factors [60,61].

The functional interpretation of the pharyngeal jaw apparatus has changed over time. It was originally hypothesized that the shift of insertion of the *levator externus 4* from the epibranchials to the LPJ makes adduction of the later possible [48]. Later it was discovered that the *levator externus muscle 4*, although inserting on the epibranchials 4, was already able to adduct the LPJ due to a coupling of the ceratobranchials 4 and the LPJ. The shift of insertion instead allows the *levator externus 4* to act on the LPJ without the concurrent use of other muscles stabilizing the ceratobranchials 4-LPJ coupling, and to avoid suboptimal rotations of the UPJ by concentrating the force only on the LPJ [50].

Here, the forces present in the pharyngeal jaw apparatus of two cichlid species are compared during ontogeny using devFEA to map the relevant biomechanics throughout development, including stress on structural elements, alterations in muscle force orientations, and the change of these parameters over the embryonic period. Standard measurements of the craniofacial morphology are given for reference and their impact on changes in pharyngeal jaw apparatus parameters is discussed. In addition, it is demonstrated how the staining and scanning of embryos can expose previously unreported structures.

## Materials & methods

### Specimens

Two species of Cichlidae were obtained with the help of a cichlid specialist in Vienna, Austria (Zierfische Aquarium). The first species, *Haplochromis elegans*, was selected due to extensive previous research [60,62–67] that helped to narrow the time window needed for observation and provided a better understanding of pharyngeal jaw apparatus development. It is a mouth brooding species and has a generalized cranial structure for Haplochromids [68,69]. Specimens were collected from two consecutive spawning from the same parental pair. Fry from the first spawnings were permitted to leave the buccal cavity naturally at 17 days post fertilization. This prevented the female from enduring stress that may have compromised future spawnings. Fry from the second spawning were manually stripped and placed in an egg tumbler, located in the tank containing the parents, until 17 days post fertilization. Any damaged or unfertilized eggs were removed to prevent mold that may have infected healthy eggs. At 17 days post fertilization, all specimens were released into the paternal tank.

The second species was *Amatitlania nigrofasciata*. It was selected as a substrate brooder for comparison with the mouth brooding *H*. *elegans*, and for the ease of breeding and extensive literature in the fish breeding community. As *A*. *nigrofasciata* are substrate brooders, each spawning was provided appropriate rock shelters. All fry were left together with the breeding pair, since *A*. *nigrofasciata* perform parental care and help feed the fry after yolk sac absorption.

All fish were kept under the same conditions with a 16:8 day:night interval in 25°C water and had a diet consisting of flakes from a local cichlid breeder. Both tanks were hooked up to the same central filtration system to ensure that the water quality remained consistent for both groups, with a UV light attached to the filtration unit in order to prevent infection. 2 fry were euthanized per day using MS-222, the standard form of euthanasia for fish according to European Union directive 2010/63/EU and Austrian animal experimentation legislation TVG 2012 which sedates the fish and then provides an anesthetic overdose.

Three *H*. *elegans* (10, 17, and 24 days post fertilization) and three *A*. *nigrofasciata* specimens (6, 11, and 19 days post fertilization) were selected from the euthanized set for use. Three sets of measurements were taken from each of the six specimens, which are described in more detail below. The first set includes UPJ size and standard cranial measurements, the latter includes: snout length (tip of the snout to the eye), head width (at widest point), head length (tip of the snout to the furthest edge of the operculum), head depth (at the operculum), and volume of the upper pharyngeal jaws. Two scans had a narrow field of view that made measurements impossible (*A*. *nigrofasciata* at 19 days post fertilization and *H*. *elegans* at 17 days post fertilization). Measurements are provided from a sibling of the same spawning. Similarly, head depth for *A*. *nigrofasciata* at 11 days post fertilization was unable to be measured due to a slight compression of the soft tissue along the dorso-ventral axis. This measurement is also provided by a sibling fish. The second set of measurements focuses on muscle orientation based on insertion and origin areas, detailed below, to describe how force vectors change during ontogeny, with changes described in terms of rotations along the rostro-caudal axis (RCA), the dorso-ventral axis (DVA), and the medio-lateral axis (MLA). The last set consists of the biomechanical quantities present in the apparatus, including the stress on the UPJ during feeding, force applied on the neurocranium from total isometric muscle contraction, and bite force.

### Finite element modeling

The highly irregular shapes found in biological systems makes it impractical to manually construct a finite element model using predefined shapes as in engineering [27], and this is particularly true during ontogeny. Instead, microCT or microMRI scans can be used to create series of image stacks that act as templates for building the finite element models. The microCT system from Xradia (model MicroXCT, Xradia, Inc., Concord, Ca, USA) at the Department of Theoretical Biology at the University of Vienna, Austria, was utilized for the present study. Image stacks from CT scans are commonly used by biologists working with FEA, however developing organisms are often not X-ray opaque enough to provide proper contrast. Therefore, depending on the specimen, it is beneficial to use a radiopaque stain. Before scanning, the embryos were quickly stained with 1% iodine in ethanol for 15 minutes [70] in order to make soft tissue X-ray dense, then returned to 70% ethanol to avoid shrinkage. The specimens were then individually mounted inside micropipette tips, with care taken to ensure the head was not tightly packed in which could cause deformation [71]. The tips were filled with 70% EtOH and sealed so no evaporation could occur, and attached to the proprietary mount for the Xradia machine. Scans consisted of 4 to 5 radiographs per degree for 181° of rotation. The reconstructed images had a voxel resolution of 2 μm^3^, depending on the focal length and X-ray source position. Images were reconstructed using the Xradia software included with the microCT system.

**Fig 2:**
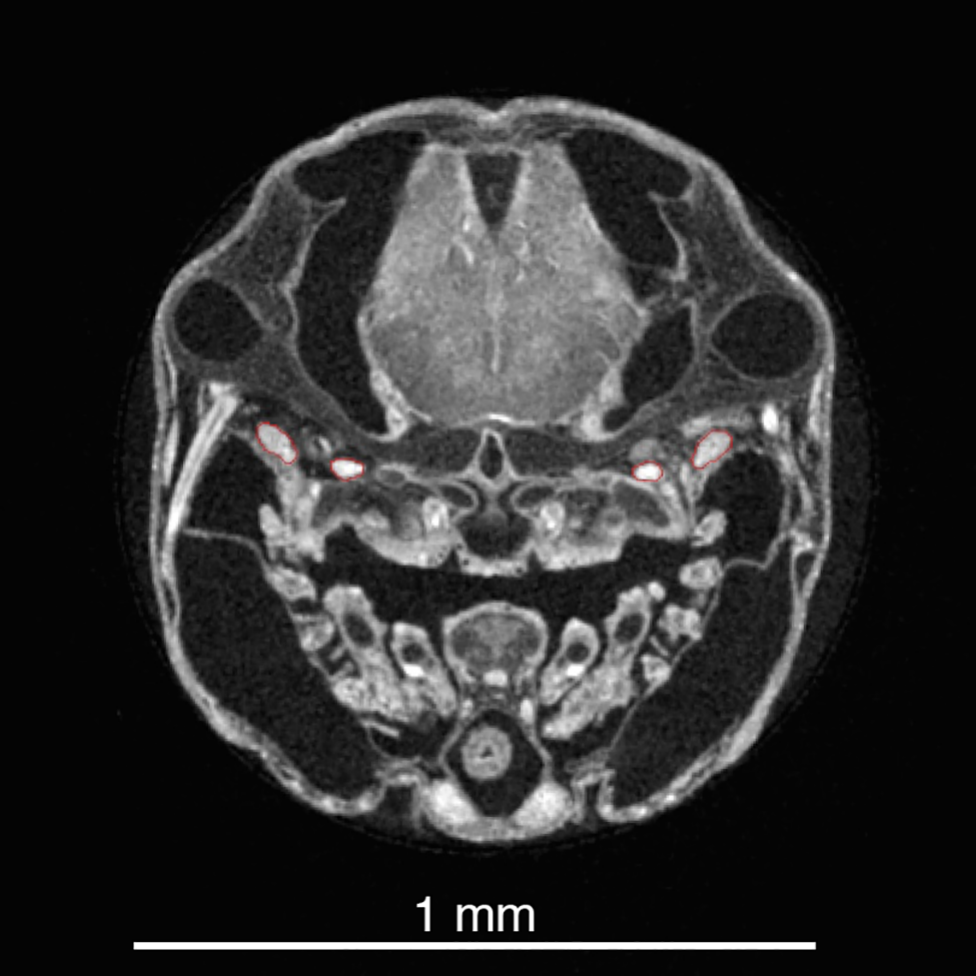
Transverse section of *A*. *nigrofasciata* at 6 days post fertilization. Note the grey scale for the upper pharyngeal jaws and skull has a large range. Muscles overlap this range, preventing the use of voxel intensity to determine tissue. All structures of interest come into contact with similar intensity voxels, which causes the selection by automatic tools such as the "magic wand" to leak into adjacent areas and make them unreliable. Instead, two muscle groups (*levator externus 4* and *levator internus lateralis*) are segmented manually here as shown by their red outlines, and this process is repeated for each structure through all images from each scan.

**Fig 3:**
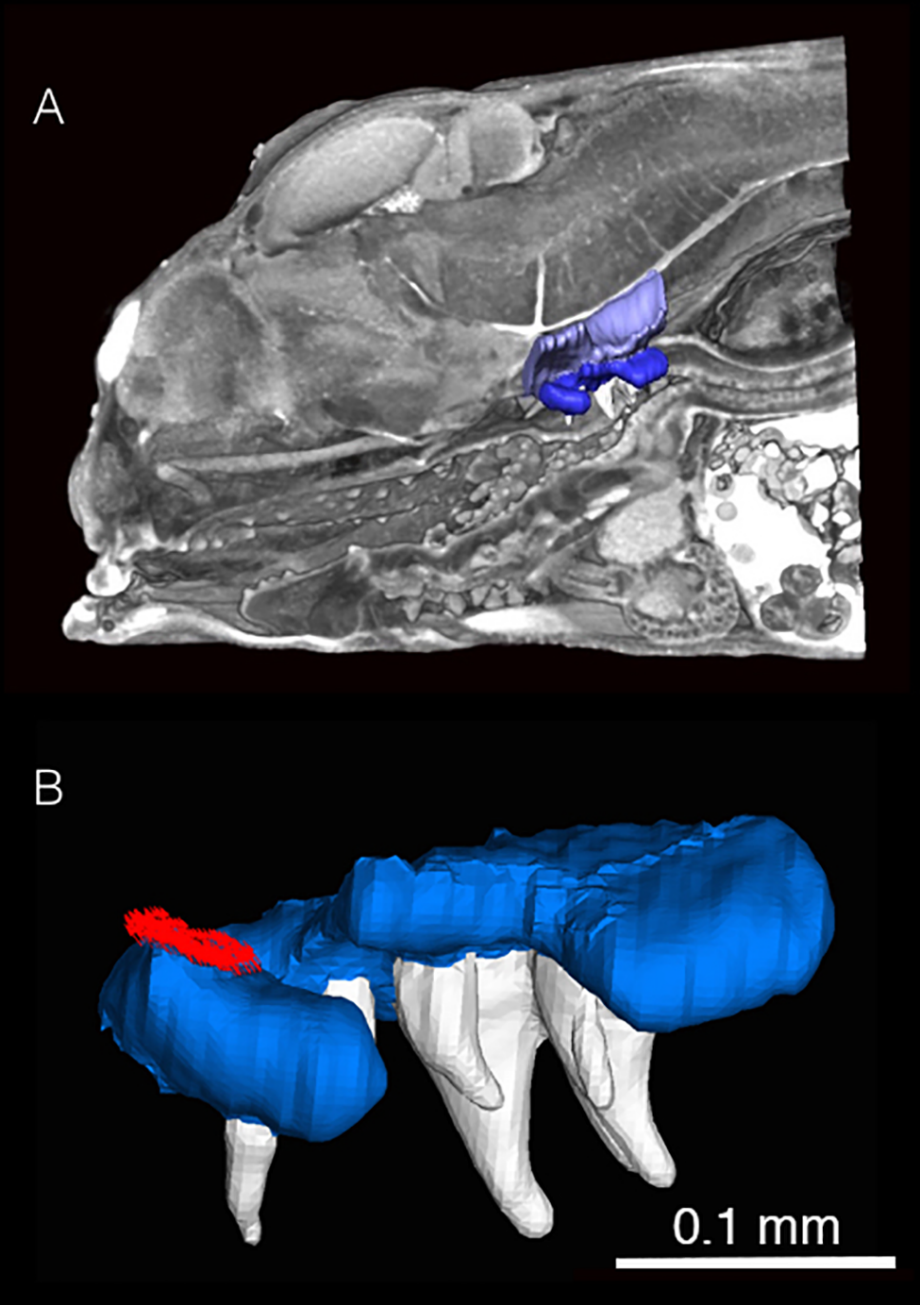
A) Volume rendering of *A*. *nigrofasciata* at 6 days post fertilization, lateral view. The upper pharyngeal jaws (blue), the portion of the neurocranium (light purple), the pharyngeal teeth (white) are shown as surface renderings that demonstrate their placement within the cranium. B) Finite element model of the upper pharyngeal jaws and teeth of *A*. *nigrofasciata* at 6 days post fertilization, lateral view. Jaws are light blue, teeth are white, and force vectors are the field of tightly packed red arrows. Arrows are the applied loads simulating the *levator internus medialis*.

Surface models were created from the selected structures. In developing organisms, particularly when contrast agents are used to make soft tissues visible, voxel intensity is poor at distinguishing structures, because dissimilar materials may have similar gray scale values, and the range of values within a single tissue may be relatively large compared to between tissue values (Fig 2). Interpolation between images or “magic wand” tools can aid in labeling, but the soft tissues of developing organisms contain many overlapping areas with similar grey scale values that make using voxel intensity or magic wand tools impractical as an approach for the whole organism. A more accurate method of tissue segmentation is to manually label fields in each image of the stack, with each field corresponding to a particular structure of interest. This is performed through highlighting relevant areas in each individual section. In the present study, the imaging software (Amira) (http://www.fei.com/software/amira-3d-for-life-sciences/) was used to label the UPJ, teeth, the contact area with the neurocranium (Fig 3), and sixteen muscles (Table 1), including their insertion and origin points. Muscle cross sectional area can be obtained using an oblique slice tool with the muscle fibers as a physiological guide, and the muscle area on the slice can then be computed by the program. Labeling the muscles allowed not only the physiological cross-sectional area to be determined, but also to use the attachment points for precise force vector orientation and placement. Muscle forces originating from the *levator externus 4* and *levator posterior*, which act on the unlabeled LPJ, were assumed to be fully transmitted to the UPJ during biting. It is possible that muscles experienced some shrinking, though this was minimized by using 70% EtOH and represent the lower end range of the muscles *in vivo* [72]. Further testing for the higher end (e.g., using water based Lugol’s solution to stain) would help establish the high end of the range and would be a good candidate for future research.

**Table 1:** Adduction muscles of the pharyngeal jaw apparatus. All muscles directly inserting on the upper pharyngeal jaws are listed, even though some do not adduct against the neurocranium, as they influence deformation and stress. The *levator externus 4* and *levator posterior* are listed, as they adduct the lower pharyngeal jaw against the upper pharyngeal jaws.

Adduction Muscles of the PJA
left / right *levator externus 4*
left / right *levator posterior*
left / right *retractor dorsalis*
left / right *levator internus medialis*
left / right *levator internus lateralis*
left / right *obliquus dorsalis*
left / right *transversus dorsalis anterior 1*
*transversus dorsalis anterior 2*
*transversus dorsalis posterior*

Once the labeling fields are completed, Amira can generate a surface mesh composed of polygons connected at their nodes. In order for the TetraGen Module of Amira to create a model composed of 3D tetrahedral elements from the surface mesh, the selected surfaces must meet a set of requirements, such as non-intersecting surface faces and a watertight continuity that contains no holes leading to the inside. The (Amira) software includes automatic surface repairs to help repair any problem nodes or surfaces. Due to processor and program constraints for both the imaging and FE software, our models were simplified to roughly 200,000 surface triangles before converting them into tetrahedral elements. In order to maximize the number of tetrahedrons used to create the model, while still maintaining the necessary muscle attachment point information, the inner sections of the muscles were deleted after taking cross sectional area measurements, leaving only the precise attachment points before conversion to a single model composed of tetrahedral elements.

After a finite element model has been created, it needs to be imported into finite element software such as the freeware (FEBio) suite (http://febio.org/febio/) or the commercially available (Strand7) (http://www.strand7.com). These programs are typically divided into three components: A Pre-Processor that includes the ability to assign material properties, loads, and restraint conditions; a Solver component that runs the simulation to calculate the resultant forces; and a Post-Processor for visualization of the results. (Strand7) was used for its ability to handle large models and the modular representation of the results, which permits any combination of muscle force vectors to be analyzed or modified. The Young’s Modulus in fish bone has a range of 3.67–8.40 GPa [73], with the lowest end of the range used in the current study since the specimens examined were not adults but the entire UPJ was covered in bone. The teeth were assigned a Young’s Modulus of 70 GPa, in order to simulate young teeth based on previous research [74]. The samples used were fixed in neutral-buffered formalin (10% NBF) and stored in 70% EtOH [71], making direct measurements of material properties impossible. However, taking material properties from similar species is well established [19,35,75,76], including for cichlid pharyngeal jaws [51]. Furthermore, the same material properties were used in each specimen and specimens were compared against models altered to represent ancestral phenotypes while holding material properties constant, allowing us to focus on differences in morphology resulting purely from altered shape. While this may have some impact on deformation, the location of stress remains the same [77]. However, flash freezing specimens after euthanasia would be preferred. Both structures were given a Poisson’s ratio of 0.3, which is commonly used in jaws and teeth [27,51,78].

**Fig 4:**
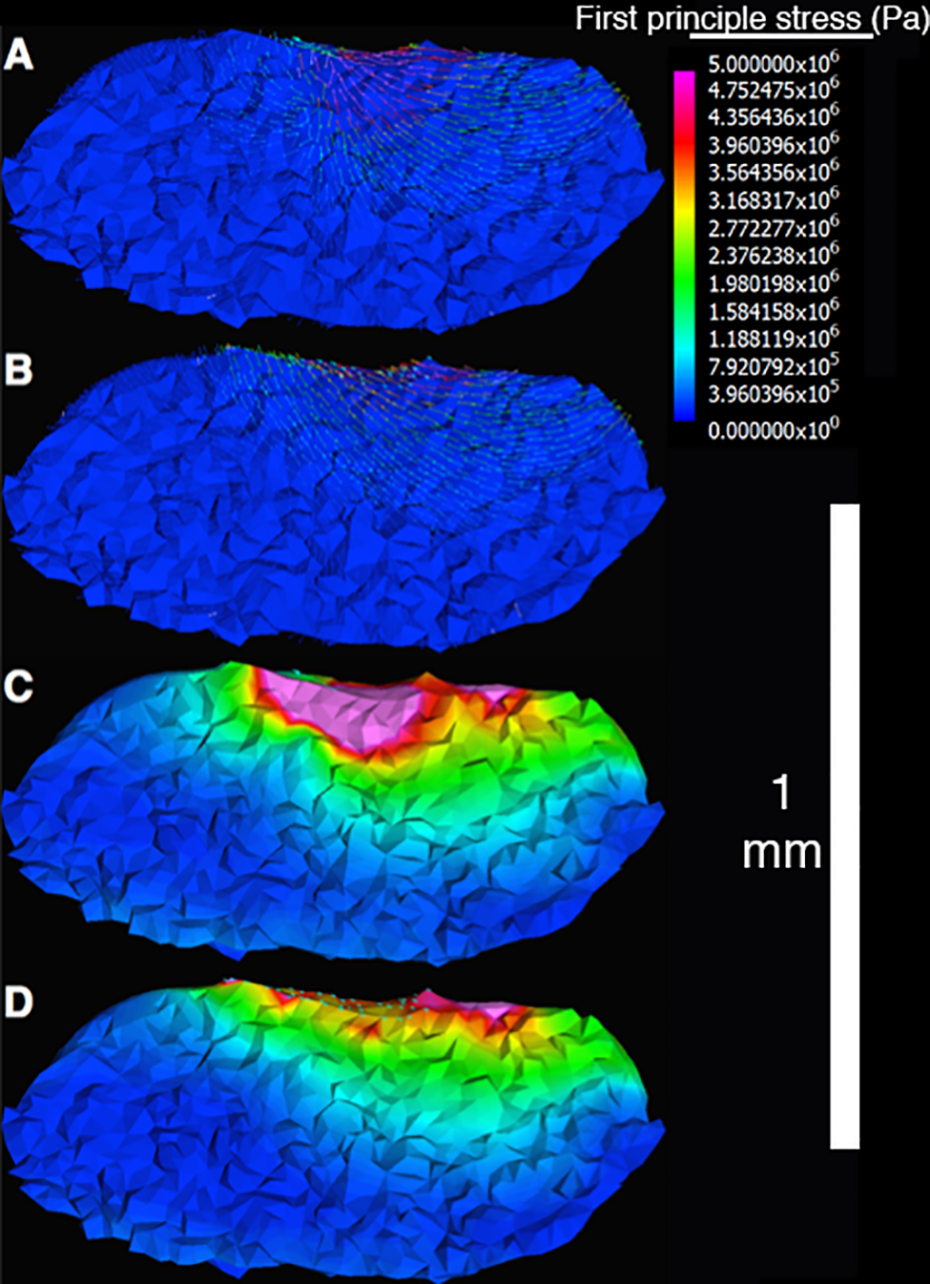
Comparison of muscle simulations using one node vs a set of nodes. The left upper pharyngeal jaw is shown in the lateral view, with a portion cut away to reveal the inner stresses. Rostral is to the left, caudal to the right. A & B, First principal stress is expressed as a set of colored vectors. A) Muscle force is applied to a single node. Note the depth the higher stress values reach and how the vectors converge from both the rostral and caudal direction. B) Muscle force is applied to a set of nodes corresponding to the muscle insertion. The higher stress values are much shallower compared to 4A, and the vectors sweep further forward from the caudal end. C & D, Von Mises Stress represented by colored bricks. C) Muscle force is applied to a single node. D) Force is distributed over the area of muscle insertion. Stress at a distance from the applied force is indistinguishable in the two cases, but closer to the load in C, since the use of only one node causes exaggerated stress levels.

Force vectors simulating muscle contractions were added based on contact between the muscles and the pharyngeal jaws. Muscles segmented in (Amira) were converted to tetrahedrons concurrently with the UPJ and teeth and imported to finite element software. The simultaneous conversion of muscle insertions and structural elements into tetrahedrons creates models with shared nodes between the two groups. The difference of the averaged nodal coordinates for muscle insertion and origin provides an accurate orientation for the force vectors. Magnitude was set for each muscle using the physiological cross-sectional area, averaged between the left and right sides for each pair, and a specific tension of 2.5 N * mm^-2^ taken from the literature for pharyngeal jaw muscles [79]. The total magnitude was then divided among the number of insertion nodes on the pharyngeal jaws, such that if a muscle produced 100 μN of force and was spread across 200 nodes, each node would have a force vector with 0.5 μN. This is a more realistic way to model the nodal forces, rather than applying the force to a single node or small number of nodes in the area of muscle attachment, as it more accurately simulates the distribution of force over the attachment area and uses the precise areas affected by each muscle. Assigning force to a single node results in large differences in the immediate area compared to multiple nodes across the attachment face, though this difference levels off as the distance to the force increases (Fig 4). The nodes shared between the neurocranium and UPJ, representing the area of contact between the two units, were fixed. This permitted the calculation of the force exerted on the neurocranium while keeping the sum of all model forces at 0 to prevent the model from unrealistic drifting.

Simulations were run using linear static analysis in (Strand7), which assumes that the materials of the structure remain linearly elastic, the displacements / deflections are negligibly small, the boundary conditions are pre-defined and will not change after load is applied, and the magnitude and direction of forces does not change over time [80]. When the Solver component for linear static is run, it creates a linear system of equilibrium with the following equation:

[K]{d}={P} Eq 1

Where [K] = Global stiffness matrix, {d} = Unknown nodal displacement vector(s), and {P} = Global equivalent nodal load vector(s)

Analysis for each condition of each model was completed in two steps. An initial analysis was performed with muscles at full force. All muscles were assumed to act simultaneously, and any non-linear effects during muscle contraction were disregarded. This permitted the establishment of the maximum bite force, stress on the UPJ, and force on the neurocranium. However, in reality some of the computed reaction forces are in the anterior-posterior direction, representing a small amount of sliding by the UPJ along the neurocranium. To minimize this so that each model did not contain any anterior-posterior sliding and instead simulated a more pure compression against the neurocranium, the force from one muscle pair was reduced using the following equation:

$$X_{}=\left(F_{total}{\text{-}}_{}F_{old}\right)/F_{old}.$$ Eq 2

Where X = the reduction factor and must be between 0 and 1, F_total_ = Force of all muscles for a given condition, F_old_ = Force of the muscle pair to be reduced.

The linear load combinations were then run using the adjusted values, and the force normal to the neurocranium was calculated. Total compressive force on the neurocranium during static compression was determined with this adjustment. Bite force, which benefits from shearing and does not require static compression, and von Mises stress were assessed without reduction. Outliers were selected by visually on a case-by-case basis to see if they were caused by the model configuration, such as corners.

Steps for devFEA:

Generalized summary of a FEA time series model generation, with devFEA methods from the present study italicized:

1. Use multiple specimens within a species to cover relevant developmental stages of the structure(s) of interest. If more than one species is used, samples between species should correspond to similar developmental events instead of enumeration of days post fertilization.
2. *Treat specimens with a radiopaque stain (e*.*g*., *iodine or PTA) to make soft tissues visible*.
3. *Label muscles and their associated attachment points along with the relevant structures as a single surface mesh*. *Muscle labeling fields should contact structures*, *even if a muscle inserts on a structure that is unnecessary for the final model*.
4. *Record the physiological cross-sectional area of each muscle*. *Afterwards*, *delete the inner portion of the muscle (i*.*e*., *all of the muscle except a layer where it contacts an attachment point)*.
5. Generate a surface mesh from the label fields. Remove problems such as holes or intersections of surface triangles. Convert the mesh into a tetrahedral volume and export to a FEA software package. This may require the file type to be converted.
6. *Average the nodes shared between a muscle and a structural element at each origin and insertion area*. *Take the difference between these two averages to determine the force vector orientation*. *Use the muscle’s physiological cross-sectional area and specific tension to calculate the force vector magnitude*. *Divide this vector equally between the insertion nodes*. *Remove muscle tetrahedrons*.
7. Assign material properties based on experimental data. Restrain a subset of the nodes to simulate realistic limitations on movement.
8. Run the appropriate solver (linear static, transient dynamic, etc.).
9. Assess relevant biomechanical properties (force, stress, strain, deformation, etc.) throughout ontogeny in relation to associated developmental events to give insight into how and where mechanotransductive pathways are elicited, the role of development in shaping evolutionary trajectories, and the more typical functional aspects of FEA.

## Results

### Standard cranial measurements and upper pharyngeal jaw size

All measurements taken from microCT scans of *A*. *nigrofasciata* and *H*. *elegans* are listed in Table 2.

**Table 2:** Cranial and upper pharyngeal jaw measurements. All measurements were taken from microCT scans.

*A*. *nigrofasciata*			
**Days Post Fertilization**	**6**	**11**	**19**
**UPJ Volume (mm**^**3**^**)**	0.0031	0.0063	0.0123
**Snout Length (mm)**	0.3	0.38	0.4*
**Head Width (mm)**	1	1.06	1.2*
**Head Length (mm)**	1.3	1.69	2.2*
**Head Depth (mm)**	1.17	1.5*	2*
***H*. *elegans***			
**Days Post Fertilization**	**10**	**17**	**24**
**UPJ Volume (mm**^**3**^**)**	0.0074	0.009	0.0164
**Snout Length (mm)**	0.42	.5*	0.57
**Head Width (mm)**	1.49	1.7*	1.85
**Head Length (mm)**	2.22	2.7*	2.98
**Head Depth (mm)**	1.68	2.2*	2.63

* Measurements taken from sibling fish.

### Muscle orientation changes during ontogeny

**Fig 5:**
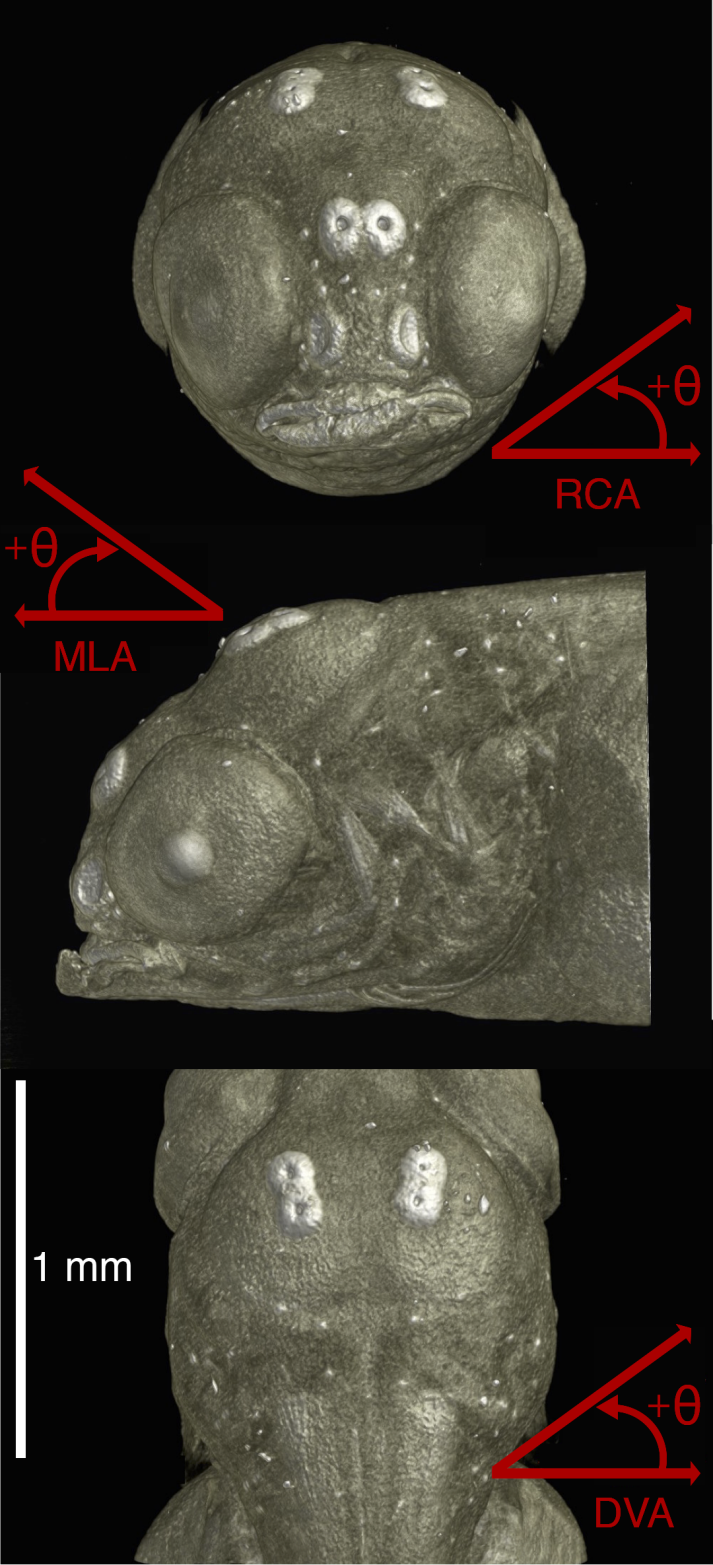
Reference rotational angles for the description of muscle orientation changes during ontogeny. RCA = rostro-caudal axis. MLA = medio-lateral axis. DVA = dorso-ventral axis. Arrows indicate directions of positive angle increase.

Isometric growth of the head, and the neurocranium in particular, results in a shifting of muscle contractions angles. These differ between species, as *A*. *nigrofasciata* has a more rounded head shape during early development compared to *H*. *elegans*, which is more elongated along the dorso-ventral axis. In both species, there is a trend to more dorsally oriented muscle angles as the cranial apophysis grows, which lowers the UPJ, and the attachment points move dorsally as the growing skull expands and the edges flex dorsally. The major changes during development of each muscle are described below for both species in terms of rotations along the rostro-caudal axis (RCA), the medio-lateral axis (MLA), and the dorso-ventral axis (DVA) (Fig 5). A full list of changes is provided in Table 3.

**Table 3:** Changes in muscle orientation. Angles are given in three axes: The rostro-caudal axis (RCA), the medio-lateral axis (MLA), and the dorso-ventral axis (DVA) (see Fig 5). The RCA ranges from 0° (lateral) to 90° (dorsal). The MLA ranges from 0° (rostral) to 90° (dorsal) to 180° (caudal). The DVA ranges from 90° (rostral) to 0° (lateral) to -90° (caudal). All measurements are given in degrees, averaged between the muscle pairs. Changes in the *levator posterior* along the DVA are large, but the muscle is oriented in this axis and the effect of the change is small.

*A*. *nigrofasciata*			
***levator externus 4***	**6 dpf**	**11 dpf**	**19 dpf**
RCA (°)	42	61	69
MLA (°)	19	35	44
DVA (°)	69	69	70
***levator posterior***	**6 dpf**	**11 dpf**	**19 dpf**
RCA (°)	81	85	89
MLA (°)	77	96	99
DVA (°)	56	-54	-84
***retractor dorsalis***	**6 dpf**	**11 dpf**	**19 dpf**
RCA (°)	86	82	87
MLA (°)	137	142	144
DVA (°)	-86	-84	-88
***levator internus medialis***	**6 dpf**	**11 dpf**	**19 dpf**
RCA (°)	19	16	23
MLA (°)	48	57	69
DVA (°)	17	11	9
***levator internus lateralis***	**6 dpf**	**11 dpf**	**19 dpf**
RCA (°)	9	35	41
MLA (°)	6	27	32
DVA (°)	56	53	54
***H*. *elegans***			
***levator externus 4***	**10 dpf**	**17 dpf**	**24 dpf**
RCA (°)	60	67	69
MLA (°)	31	35	35
DVA (°)	71	73	75
***levator posterior***	**10 dpf**	**17 dpf**	**24 dpf**
RCA (°)	87	89	88
MLA (°)	92	87	86
DVA (°)	-31	69	69
***retractor dorsalis***	**10 dpf**	**17 dpf**	**24 dpf**
RCA (°)	80	89	89
MLA (°)	137	133	131
DVA (°)	-81	-89	-88
***levator internus medialis***	**10 dpf**	**17 dpf**	**24 dpf**
RCA (°)	22	26	30
MLA (°)	65	68	53
DVA (°)	11	11	24
***levator internus lateralis***	**10 dpf**	**17 dpf**	**24 dpf**
RCA (°)	30	41	43
MLA (°)	20	30	29
DVA (°)	57	57	59

#### Levator externus 4

The *levator externus 4* inserts on the LPJ to create the new muscle sling, shifting dorsally during ontogeny in both species. In *A*. *nigrofasciata*, it changes from 42° to 69° in the RCA, and from 19° to 44° in the MLA. In *H*. *elegans*, the muscle shifts from 60° to 69° in the RCA, and 31° to 35° in the MLA.

#### Levator posterior

In *A*. *nigrofasciata*, the *levator posterior* moves to a more dorsal and caudal angle. In the RCA it shifts from 81° to 89° (more dorsal), and in the MLA from 77° to 99° (more caudal). By contrast, in *H*. *elegans* the *levator posterior* becomes oriented more to the rostral, shifting from 92° to 86° in the MLA.

#### Retractor dorsalis

The *retractor dorsalis* exhibits a general trend towards the caudal in both species. In *H*. *elegans*, it shifts from 137° to 131° in the MLA. There is also a dorsal change from 80° to 89° in the RCA that later holds steady between 17 and 24 days post fertilization. In *A*. *nigrofasciata*, the caudal change is from 137° to 144° in the MLA.

#### Levator internus medialis

The *levator internus medialis* rotates to a more lateral and dorsal position in *A*. *nigrofasciata*. The DVA shifts from 17° to 9° (more lateral), and the MLA changes from 48° to 69° (more dorsal). The opposite is true in *H*. *elegans*, where rotation in the DVA is from 11° to 24° (more medial and rostral), and from 65° to 53° in the MLA, a shift away from the dorsal.

#### Levator internus lateralis

The *levator internus lateralis* has a dorsal shift in both species. In *A*. *nigrofasciata*, it has a dorsal change from 9° to 41° in the RCA, and another dorsal rotation from 6° to 32° in the MLA. In *H*. *elegans*, there is a dorsal shift from 31° to 43° in the RCA.

### Force on the neurocranium

**Fig 6:**
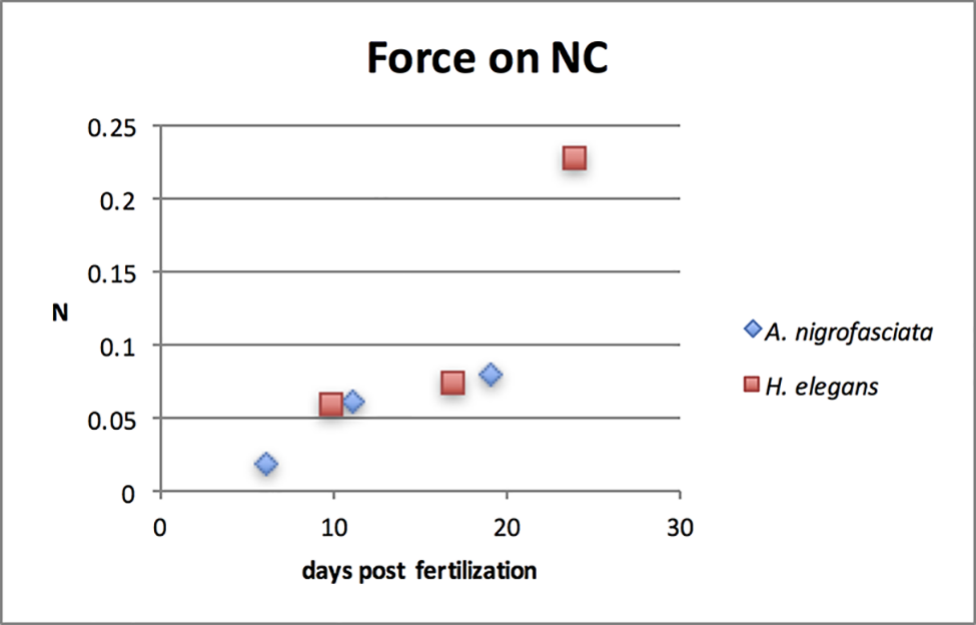
Force on the neurocranium (NC) from the upper pharyngeal jaws in Newtons (N) in *H*. *elegans* and *A*. *nigrofasciata*. Force in both species is comparable based on days post fertilization. Since *H*. *elegans* has a longer period with the yolk sac (17 days post fertilization) compared to *A*. *nigrofasciata* (11 days post fertilization), the former experiences a large increase in force directly after yolk sac absorption that is not seen in *A*. *nigrofasciata*.

Force on the neurocranium from the UPJ due to total isometric adductor muscle contraction in the pharyngeal jaw apparatus is reported in Fig 6. The two species experience comparable force on the neurocranium in relation to days post fertilization. However, *H*. *elegans* experiences yolk sac absorption later than *A*. *nigrofasciata* (day 17 compared to day 11), showing that the force on the neurocranium follows a similar pattern between species’ development time instead of developmental stage.

### Bite force

**Fig 7:**
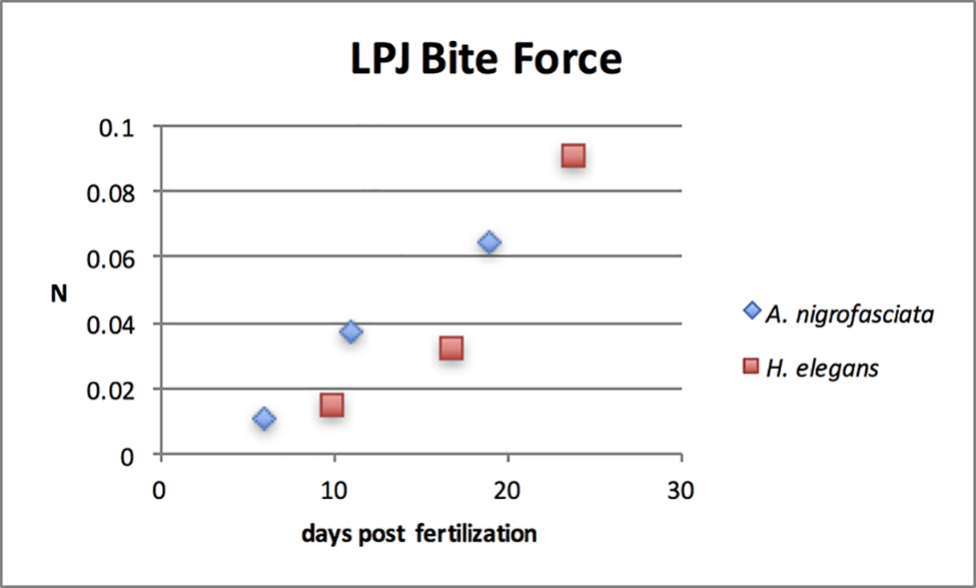
Adduction force of the lower pharyngeal jaw from the *levator externus 4* and *levator posterior* muscles in *H*. *elegans* and *A*. *nigrofasciata*. While the total force always increases over time for both species, the rate of change increases between pre and post yolk sac absorption in *H*. *elegans* yet decreases during the same period in *A*. *nigrofasciata*.

Fig 7 displays the adduction force of the LPJ from the *levator externus 4* and *levator posterior* muscles. *A*. *nigrofasciata* has an increase in bite strength of 337% between the 6 and 11 days post fertilization specimens. This rate then decreases to an increase of 171% between the 11 and 19 days post fertilization specimens. *H*. *elegans*, on the other hand, has an increase in bite force rate after yolk sac absorption. The force begins with an increase of 223% between the 10 and 17 days post fertilization specimens. It then further increases to 289% between the 17 and 24 days post fertilization specimens.

### Stress on the upper pharyngeal jaws

**Fig 8:**
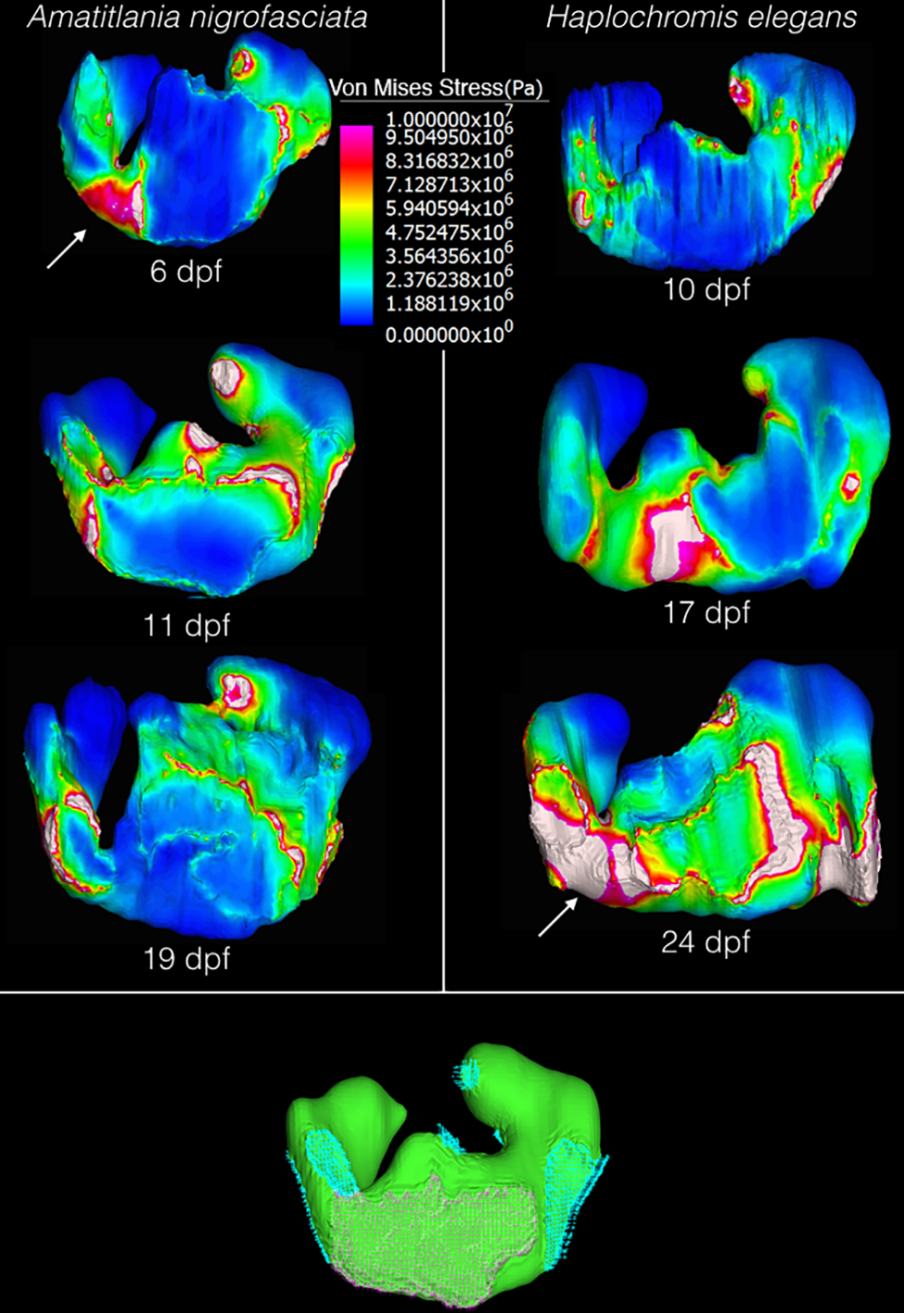
Locations of von Mises stress on the right upper pharyngeal jaw resulting from complete muscle contraction. Dorsal view of the upper pharyngeal jaw of *A*. *nigrofasciata* and *H*. *elegans*. Age increases from top to bottom. Images of the upper pharyngeal jaw are not to scale, they have been adjusted in size so they can be compared. Arrows point to the area of highest stress, where the two infrapharyngobranchials attach on *A*. *nigrofasciata* at 6 days post fertilization and on *H*. *elegans* at 24 days post fertilization. The lower image in green gives an example of the placement of boundary conditions, where the UPJ met the neurocranium (pink) and the load conditions, where the muscles act on the pharyngeal jaw (blue). dpf = days post fertilization.

The area between the two infrapharyngobranchials that comprise the UPJ experiences the highest level of Von Mises stress outside of areas directly acted on by the pharyngeal muscles or where the UPJ presses onto the skull. In *H*. *elegans*, this stress increases throughout the three periods examined. However, in *A*. *nigrofasciata* the same area undergoes decreasing levels of stress (Fig 8).

### Cement glands

**Fig 9:**
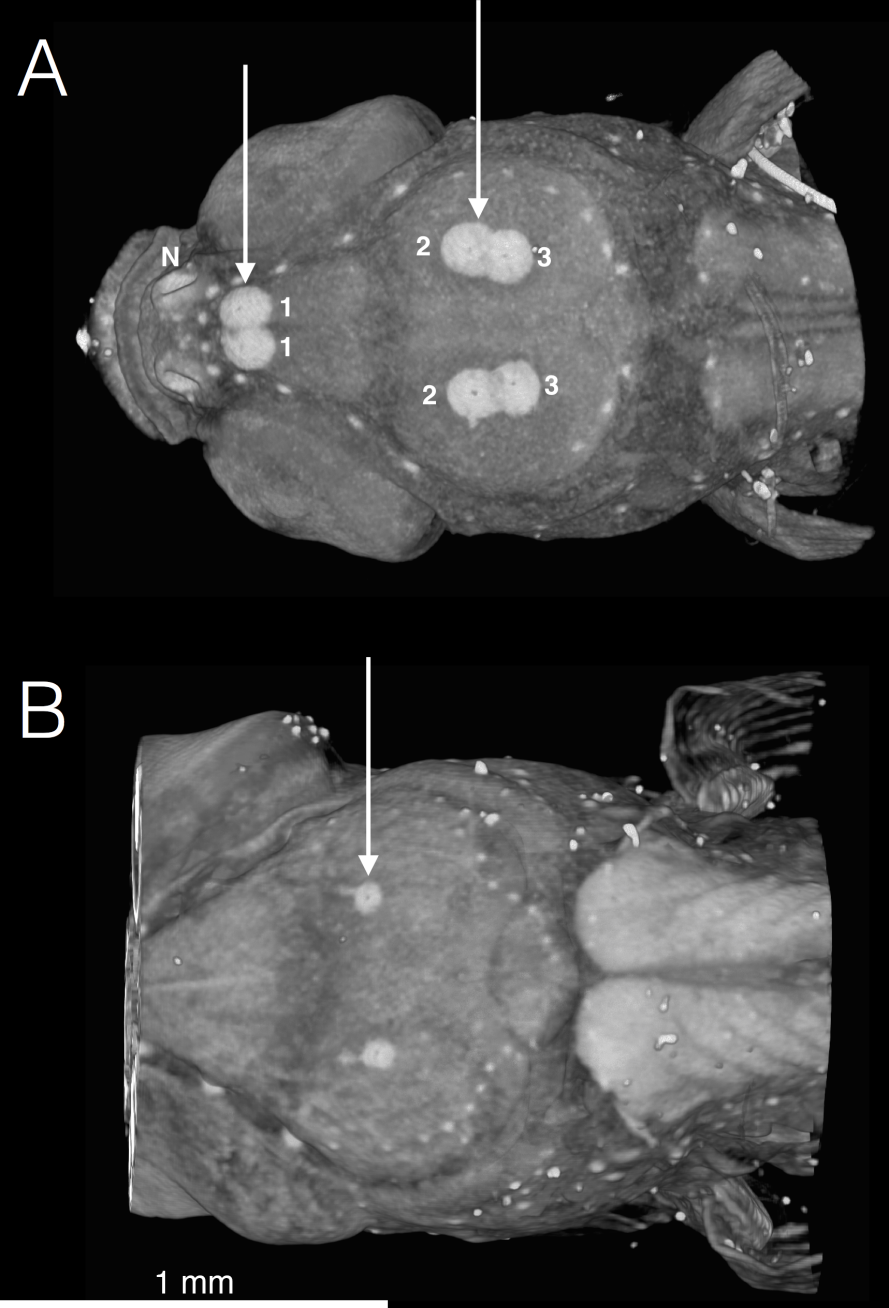
Dorsal view of *A*. *nigrofasciata* at (A) 6 and (B) 15 days post fertilization showing cement glands, labeled as pairs 1–3. Glands are used to attach the fry to the substrate between hatching (3 days post fertilization) and onset of free swimming (6 days post fertilization). The glands slowly decrease in size over time, but are still present at 15 days post fertilization. N = Nostril.

As a byproduct of the contrast technique used in the present study, cement glands not previously reported to exist in *A*. *nigrofasciata* are described. Staining for microCT scans reveals a set of three pairs of cement glands in symmetrical arrangement along the sagittal midline. The most rostral of these lie caudal to the nostrils and are close enough to the midline to touch. The caudal four glands are on top of the cranium. The second pair of glands is slightly more lateral than the third pair, and the two pairs are also close enough to be touching. These glands are present at 6 days post fertilization (the earliest scans made), and and the 3^rd^ pair is still slightly visible at 15 days post fertilization (Fig 9). They have disappeared by 19 days post fertilization.

## Discussion

The biomechanics of the pharyngeal jaw apparatus and quantified details on the positioning of the relevant tissues has been lacking from descriptions of cichlid development. This is particularly troublesome as the morphology of the apparatus is dependent on biomechanical cues. Here we used microCT imaging with radiopaque staining to create a time series of finite element models, in order to produce a detailed report on the system’s changes during development. This includes the forces present at the compression point between the UPJ and the neurocranium, the arrangement of the muscular system where the stresses occur, and functional significance from changes to the system during ontogeny. We also describe newly found structures, the cement glands, in *A*. *nigrofasciata*.

In cichlid pharyngeal jaws, shape changes from growth of the skull adjust the orientation of the acting muscles. The complex three-dimensional shape of the apparatus including its numerous muscle groups leads to directional changes that are not isometric. Furthermore, it is not possible to deduce these changes from the standard cranial measurements, while 3D imaging can accurately determine muscle orientation changes. However, the amount of variation present in muscle orientation during development requires further testing and was beyond the scope of this study. Future research on the variation in muscle placement and its functional and developmental significance could greatly benefit the field. Furthermore, skeletal growth is impacted by external factors such as nutrition and temperature, as well as physical forces altering bone shape and growth [62].

When performing FEA on small developing structures, complications exist that are not present with adult structures. This can be seen in Fig 4, where stresses reaching 5 MPa penetrate a quarter of the depth into the UPJ on models with single node loads due to the small size of the structure. This is unrealistic as it approaches the levels of muscular stress on the skull seen in adults of larger organisms during mastication. For example, adult bat skulls display approximately 11 MPa from three masticatory muscles, though thin structures or areas adjacent to single node loads experience more [27]. On models of pharyngeal jaws with the force spread across the area of insertion, the same depth experiences less than 2 MPa, and higher stresses are limited to bricks at the surface. While limited node use is not detrimental to larger adult organisms, during ontogeny this must be avoided.

Similarly, when the model utilizes single node loads, the first principal stress vectors that are within the area of insertion orient towards the stressed node, regardless of the direction of the force applied. When using the entire insertion area, these vectors orient with the direction of force. Since the number of nodes at which forces are applied impacts these stresses, and insertion areas are highly variable (from less than 50 to over 1000 nodes in the current models), it is necessary to independently identify each muscle area.

Seemingly similar structures can differ substantially in their stress-dependent behaviors over developmental time. In the present case, one area of interest is between the infrapharyngobranchials 2 and 3 (that combine to form the UPJ), since this is the weakest area of the UPJ. In *H*. *elegans*, this area experiences stress of approximately 10 MPa at 10 days post fertilization that slowly decreases throughout ontogeny, while *A*. *nigrofasciata* begins with lower stress that then increases to a large area with stress over 10 MPa (Fig 8) at 19 days post fertilization.

Initially, the force between the UPJ and the neurocranium increases in both species as they develop, and stays between 0.06 N and 0.08 N at corresponding days post fertilization (11–19 days post fertilization for *A*. *nigrofasciata* and 10–17 days post fertilization for *H*. *elegans*). But subsequently, a sharp rise in force can be seen to 0.23 in *H*. *elegans* at 24 days post fertilization. This occurs during the switch to external feeding and the development of the neurocranial apophysis upon which the basipharyngeal joint rests [60].

While data such as force on the neurocranium, muscle orientation, or force distribution can be accurately determined, the absolute value of stress may vary slightly if the material properties were off. While it is well established to use material properties obtained from other species instead of directly from the specimens examined [19,35,75,76], and this has been performed during research on cichlids [51], the actual stress values may differ slightly as the material properties were not available to be directly measured at this time. Nevertheless, the qualitative locations of the stress will remain the same [77].

Whereas FEA on adult organisms is usually used to derive information mechanical function in tasks such as feeding or locomotion, in developing organisms it is to better understand the processes that ensure proper form production. The strongest use will be in combination with programs that study how mechanotransductive processes are elicited [81,82] or impact development [11–13]. The extensive time required for manual segmentation currently hinders the number of specimens that can be examined. As new technology for image segmentation becomes available, it will be important to create large sets of models from the same days post fertilization to determine the range of muscle configurations and the relative impact they have on the development of form. Particularly in developing organisms, this would benefit highly from tissue specific radiopaque staining procedures, some of which are currently under development. Using various stains on multiple specimens or combinations of stains would be the most reliable method for obtaining unique peaks of radiopacity. More general radiopaque stains such as iodine or PTA do have other benefits, though. In the present study they highlight previously undocumented structures in *A*. *nigrofasciata*: the cement glands. While fry become free swimming at six days post fertilization, these glands persist for up to fifteen days post fertilization, possibly allowing older individuals to remain still in water without movement of the fins. Cement glands are known to be used to attach the larvae to the substrate before they are free swimming. During times of danger, young free-swimming fry often hide under overhanging rocks, and it is possible the retention of the glands during the early free swimming period is used to attach the fry to the substrate.

The added functional abilities and increased modularity have made the derived pharyngeal jaw apparatus a key innovation in the rapid speciation of cichlids [48,50]. Evolutionary change of structural components, initiated by natural selection or environmental induction, has altered the biomechanical forces present in the pharyngeal jaw system. One possible method for calculating these forces is outlined here. While this line of inquiry is in its early stages, it has the potential to connect mechanotransductive pathways in development with macroevolutionary forms of change. devFEA models can reveal when and where mechanotransductive pathways will be activated by indicating high strain areas that are potent target regions for evolutionary variance and novelty formation [83]. The addition of this kind of approach to the repertoire of evolutionary studies can create a more comprehensive understanding of the role of development in the realization of morphological form.

## Abbreviations

FEA: finite element analysis
UPJ: upper pharyngeal jaws
LPJ: lower pharyngeal jaws
RCA: rostro-caudal axis
DVA: dorso-ventral axis
MLA: medio-lateral axis
