# Correction: Developmental finite element analysis of cichlid pharyngeal jaws: Quantifying the generation of a key innovation

**DOI:** 10.1371/journal.pone.0195393

**Published:** 2018-03-29

**Authors:** The PLOS ONE Staff

The second author, Gerd B. Müller, should be designated as a corresponding author. The contact email for Dr. Müller is: gerhard.mueller@univie.ac.at. The publisher apologizes for the error.
